# Development and Validation of a Risk Stratification Model of Pulmonary Ground-Glass Nodules Based on Complementary Lung-RADS 1.1 and Deep Learning Scores

**DOI:** 10.3389/fpubh.2022.891306

**Published:** 2022-05-23

**Authors:** Qingcheng Meng, Bing Li, Pengrui Gao, Wentao Liu, Peijin Zhou, Jia Ding, Jiaqi Zhang, Hong Ge

**Affiliations:** ^1^Department of Radiology, The Affiliated Cancer Hospital of Zhengzhou University, Zhengzhou, China; ^2^Department of Radiotherapy, The Affiliated Cancer Hospital of Zhengzhou University, Zhengzhou, China; ^3^Department of Radiology, The People's Hospital of Nanzhao Country, Nanyang, China; ^4^Yizhun Medical AI Co. Ltd, Beijing, China

**Keywords:** lung neoplasms, risk stratification, convolutional neural network, lung imaging reporting and data system, X-ray computed tomography

## Abstract

**Purpose:**

To assess the value of novel deep learning (DL) scores combined with complementary lung imaging reporting and data system 1.1 (cLung-RADS 1.1) in managing the risk stratification of ground-glass nodules (GGNs) and therefore improving the efficiency of lung cancer (LC) screening in China.

**Materials and Methods:**

Overall, 506 patients with 561 GGNs on routine computed tomography images, obtained between January 2017 and March 2021, were enrolled in this single-center, retrospective Chinese study. Moreover, the cLung-RADS 1.1 was previously validated, and the DL algorithms were based on a multi-stage, three-dimensional DL-based convolutional neural network. Therefore, the DL-based cLung-RADS 1.1 model was created using a combination of the risk scores of DL and category of cLung-RADS 1.1. The recall rate, precision, accuracy, per-class F1 score, weighted average F1 score (F1_weighted_), Matthews correlation coefficient (MCC), and area under the curve (AUC) were used to evaluate the performance of DL-based cLung-RADS 1.1.

**Results:**

The percentage of neoplastic lesions appeared as GGNs in our study was 95.72% (537/561) after long-period follow-up.Compared to cLung-RADS 1.1 model or DL model, The DL-based cLung-RADS 1.1 model achieved the excellent performance with F1 scores of 95.96% and 95.58%, F1_weighted_ values of 97.49 and 96.62%, accuracies of 92.38 and 91.77%, and MCCs of 32.43 and 37.15% in the training and validation tests, respectively. The combined model achieved the best AUCs of 0.753 (0.526–0.980) and 0.734 (0.585–0.884) for the training and validation tests, respectively.

**Conclusion:**

The DL-based cLung-RADS 1.1 model shows the best performance in risk stratification management of GGNs, which demonstrates substantial promise for developing a more effective personalized lung neoplasm management paradigm for LC screening in China.

## Introduction

The detection rate of pulmonary ground-glass nodules (GGNs) has been increasing dramatically owing to the widespread use of multi-slice spiral computer tomography (CT) and CT screening programs for lung cancer (LC) detection (1). Furthermore, GGNs may be observed in benign conditions, such as focal interstitial fibrosis, inflammation, hemorrhage, and neoplasms (including atypical adenomatous hyperplasia and adenocarcinoma *in situ*, and malignancies) (2). Early diagnosis and treatment of LC through incidental detection or screening is a promising strategy for improving the detection rate of early LC and for reducing the associated mortality (3). Lung imaging reporting and data system (Lung-RADS) screening interpretation, proposed by the American College of Radiology and revised in 2019 for low-dose CT risk stratification, has been successfully used to reduce the rate of false-positives with only a small corresponding decrease in sensitivity (4). However, incidental or screening-detected LC appearing as GGNs would have either been missed or underdiagnosed by Lung-RADS 1.1, because the size of GGNs was <30 mm (14,137.2 mm^3^) (5). Moreover, the long follow-up period recommended by Lung-RADS version 1.1 increases costs, additional radiation exposure and patient anxiety owing to additional scans (6). Furthermore, the observation of pulmonary nodules by radiologists is both labor-intensive and time-consuming and the results can often be different because of personal differences. Jiang H, et al. (7) adopted the semi-automatic four-channel convolution neural networks model for detecting different types of nodules and achieved a sensitivity of 80.06% with 4.7 false positives per scan and a sensitivity of 94% with 15.1 false positives per scan, but the results of several review studies (8, 9) show that the computer-aided detection system improve the existing systems and propose new solutions because of its false positives rate and its ability to detect nodules. The Watershed and histogram of oriented gradients (HOG) techniques for distinguishing nodules and a rule-based classifier and support vector machine (SVM) for eliminating false positives were used by Firmino et al. (10) and yielded the ROC curves with areas of 0.72 for nodules with indeterminate malignancy, and a multi-view knowledge-based collaborative (MV-KBC) deep model was used to separate malignant from benign nodules in the study by Xie et al. (11) and achieved the accuracy of 91.60% for lung nodule classification with an AUC of 95.70% for different types of nodules. However, these algorithms need to be promoted further in clinical practice, and more sophisticated risk stratification and prediction models will be beneficial for appropriate management of indeterminate GGNs. Therefore, we proposed a DL-based version of complementary Lung-RADS 1.1 (cLung-RADS 1.1) to predict pulmonary neoplasms manifesting as GGNs on CT images, and therefore validated this model in actual clinical scenarios.

## Materials and Methods

### Data Source

This single-center study protocol was approved by the Affiliated Tumor Hospital of the Zhengzhou University Medical Ethics Committee (Ethics Approval Number: 2021-KY-0022). The requirement of obtaining informed consent from participating patients was waived because of the retrospective nature of this study. Database were retrospectively collected from 736 patients, with one or more GGNs detected on a thoracic CT from January 2017 to March 2021.

**Figure 1:**
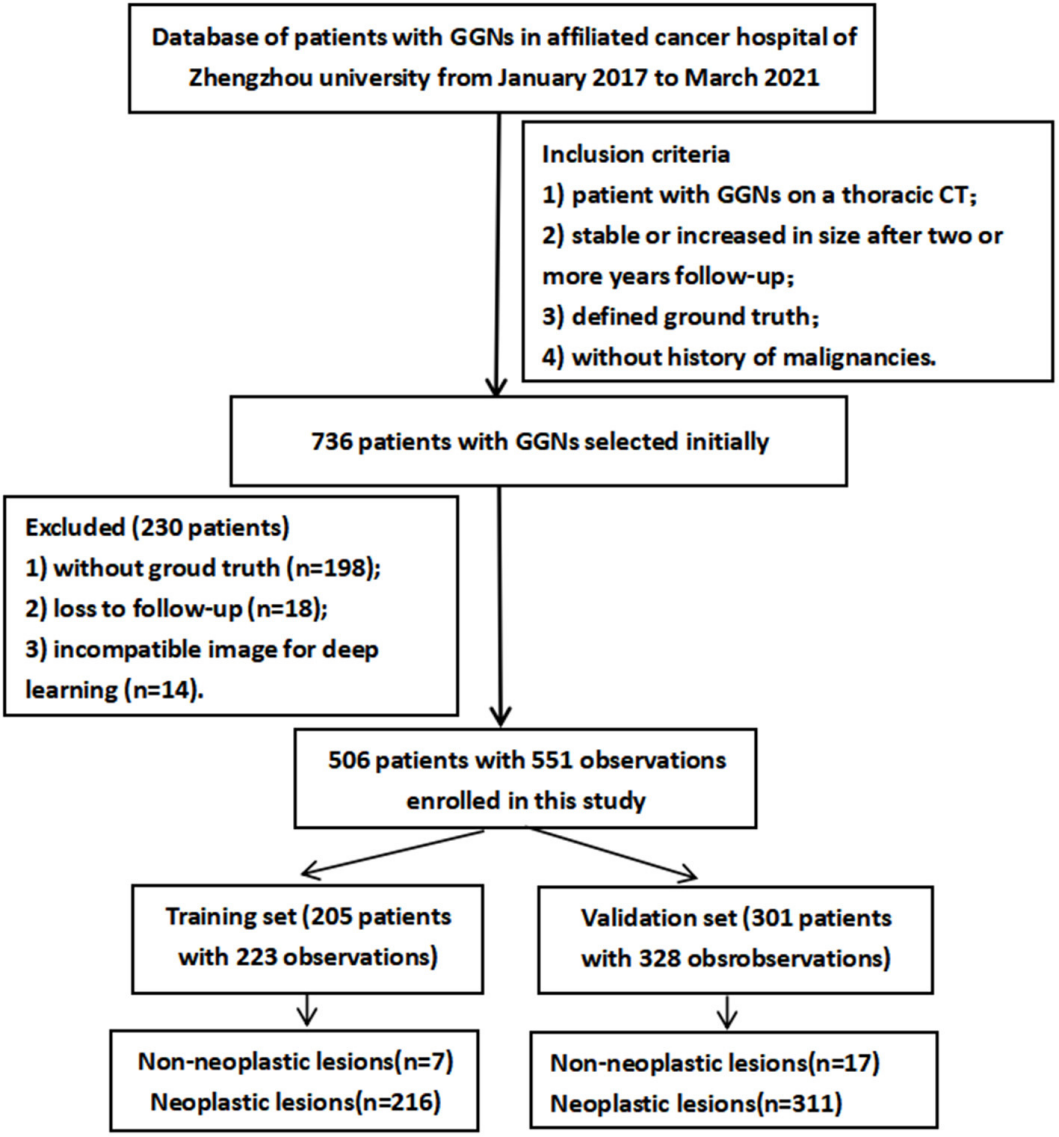
Flowchart depicting the selection of patients for this study.

The inclusion criteria were (1) patient reported as having one or more GGNs detected on a thoracic CT, (2) GGNs were stable or increased in size after follow-up at two or more years, (3) defined ground truth owing to accompaniment of clinical symptoms or severe patient anxiety, and (4) patient had no history of or currently known extra-thoracic malignancies. The exclusion criteria were (1) patients without pathological diagnoses or patients with no follow-up, (2) rejection of chest CT by DL owing to incompatible image parameters (i.e., CT slice thickness >5 mm or poor image quality), and 3) patients with lung or other site infections. The process of selecting the study population is illustrated in Figure 1. The enrolled patients were randomly divided into a training set (205 patients, 223 observations) and validation set (301 patients, 328 observations). The CT images from all cases were anonymized, and the clinical data or pathological diagnosis findings were collected.

### Image Acquisition and Quality Control

The CT examinations of all patients were performed using a multi-slice CT scanner (iCT-256, Siemens or LightSpeed-16, GE) with a tube voltage of 120 kVp and tube current of 100–300 mA. The pixel spacing of the CT images ranged from 0.625 to 0.867 mm, depending on the patient size, and the reconstruction slice thickness was 1 mm. Each CT image was reconstructed in an image matrix of 512 × 512 pixels. Unenhanced spiral acquisitions were obtained with a breath-hold from the thoracic inlet to the lung bases with images. These images were reconstructed using a standard algorithm. A non-ionic contrast agent was used for the multi-phase enhanced scanning process in 76 patients, and a high-pressure bolus was injected through the elbow vein at a rate of 1.8–2.5 mL/s. The dosage of the contrast agent was 1.5–2.0 mL/kg, and the flow was 2–3 mL/s. Each GGN on the CT, along with its multiplanar reconstruction, was independently interpreted by three thoracic radiologists with 7, 10, and 15 years of experience, respectively, they were not privy of the pathological results. Considering cases of disagreement on the cLung-RADS 1.1 categories among the three radiologists, the images were re-reviewed together, and a consensus categorization was achieved.

### Description of the DL Neural Network Process: Lung Nodule Detection and Classification

**Figure 2:**
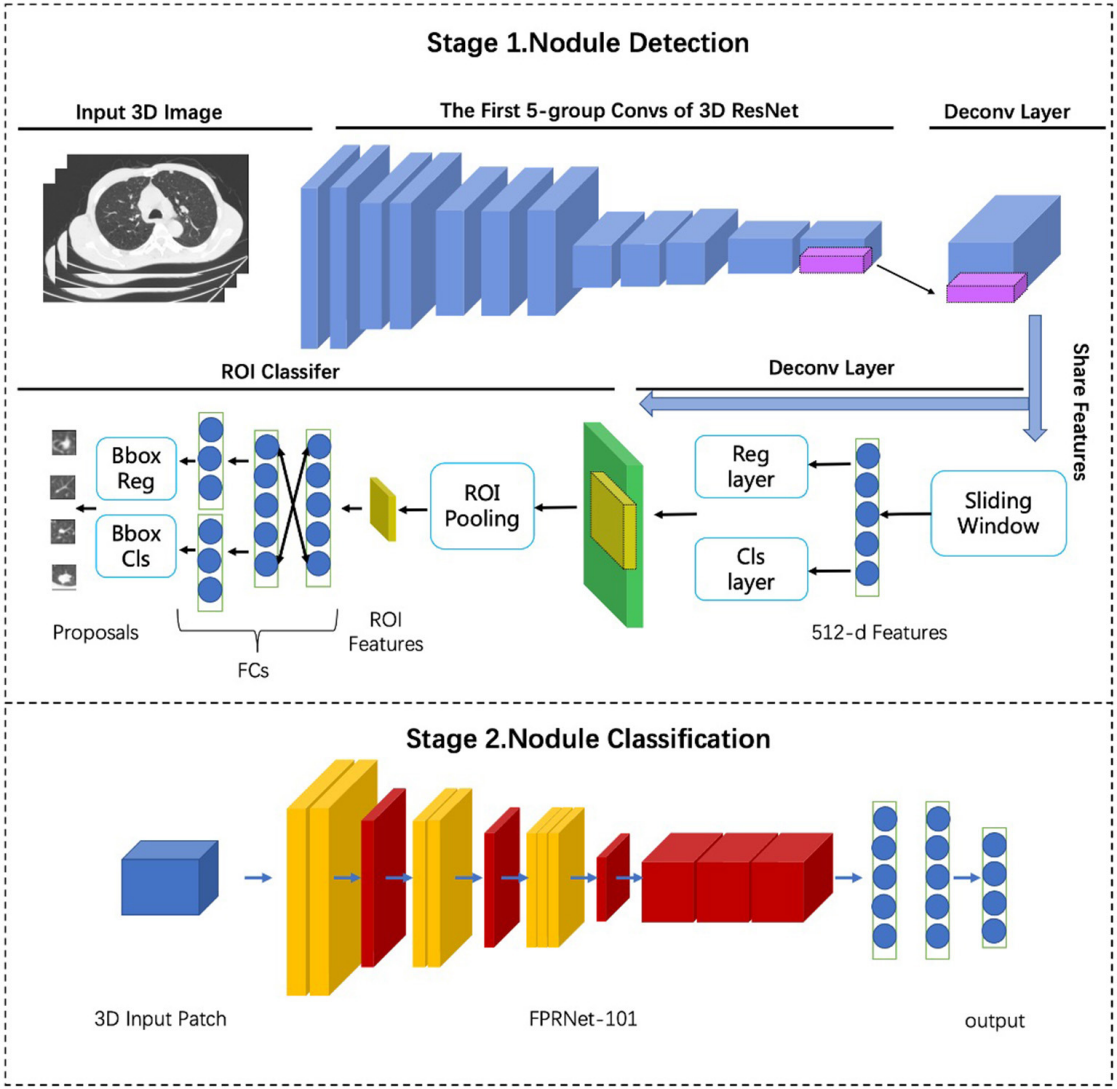
Frame structure of AI based on multi-stage 3D-DCNN algorithms.

The framework of commercial DL was based on multi-stage three-dimensional deep convolutional neural network (3D-DCNN) algorithms (12). The DL algorithm of the lung nodule diagnosis model comprises two stages (Figure 2): a nodule detection stage and nodule classification stages. The first stage extracts high-quality nodule proposals based on the 3D ResNet model and faster convolutional neural network detector, whereas the second stage employs a false positive reduction network (referred to as FPRNet-101) for precise lung nodule classification. Combining both stages, the lung nodule diagnosis model achieved state-of-the-art performance and was endowed with human domain knowledge, resulting in more precise, powerful, and understandable diagnoses. The malignancy scores of GGNs using the commercial DL approach were classified as low (<50%), medium (50–70%), and high (70–100%). Moreover, nodules with medium or high malignant risk scores were defined as positive nodules.

### Risk Stratification Management Model of Incidental Pulmonary GGNs: Progress Description of DL-Based CLung-RADS 1.1

The cLung-RADS 1.1 was redesigned according to the GGN-vessel relationships (GVR) which were categorized into four different types according to imaging features. We identified the type I of GVR and size <30 mm as Lung-RADS 2, type I of GVR and size≥30 mm or type II of GVR as Lung-RADS 2, and any sizes with GGN of type III as Lung-RADS 4a and with type IV as Lung-RADS 4b, Category 3 or 4 nodules with additional features or imaging findings that increased the suspicion of malignancy were defined as Lung-RADS 4x, detailed in **Table 2** and the reference (13), and therefore a novel DL-based cLung-RADS 1.1 model was developed to incorporate the additional information provided by the DL risk scores. A management strategy was developed to leverage the increased diagnostic accuracy achieved by the artificial intelligence (AI) algorithm. Pulmonary GGNs that were initially classified as category 3, 4A, or 4B of cLung-RADS, however, they were considered as sufficiently middle- or high-risk by the DL algorithm (i.e., with a DL risk score above the operating point defined to match the average sensitivity of the three radiologists applying cLung-RADS 1.1), were upgraded separately to categories 4A, 4B, and 4X, respectively. This was done to either reduce the follow-up period of low-dose CT (LDCT) scanning or to indicate the optimal operating time. Lung GGNs initially classified as category 3, 4A, or 4 B and deemed to be low -risk by the DL algorithm (that is, with a DL risk score below the chosen operating point) were maintained as category 3, 4A, or 4B, respectively. Pulmonary GGNs for which the cLung-RADS 1.1 classifications were considered concordant (category 2 or 4X with any DL risk score) were managed based on the initial cLung-RADS 1.1 classification. There was no change in management based on the DL-informed management strategy, shown in **Table 3**. A risk stratification of more than or equal to category 4A based on the DL-based cLung-RADS 1.1 for GGNs was defined as the neoplasm point in clinical scenarios.

### Statistical Analysis

Statistical analyses were performed using the SPSS software (version 24; IBM Corporation). Data were reported as mean ± standard deviation (SD). An independent *t*-test was used to compare the quantitative data. Counting data were described in terms of frequency and percentage, and comparisons between groups were conducted using the chi-square test. When the expected value was <1 or the pre-test probability was approximately the same as the test level, Fisher's exact test was used instead. The validity and predictive values of cLung-RADS 1.1, DL, and DL-based cLung-RADS 1.1 were calculated for the recall rate, precision, accuracy ($ACC=\frac{TP+TN}{TP+FP+TN+FN}$), per-class F1 score ($F1=\frac{2\times Precision\times Recall}{Precision+Recall}$), weighted average F1 score ($F1weighted=\frac{\left(1+\beta^{2}\right)\times Precision\times Recall}{\left(\beta^{2}\times Precision\right)\;+Recall}$), and Matthews correlation coefficient ($MCC=\frac{TP\times TN-FP\times FN}{\sqrt{\left(TP+FP\right)\left(TP+FN\right)\left(TN+FP\right)\left(TN+FN\right)}}$), respectively. Here, TP denotes true positive, FP denotes false positive, TN denotes true negative, FN denotes false negative, precision denotes the precision value ($Precision=\frac{TP}{TP+FP}$), and recall denotes the recall value ($Recall=\frac{TP}{TP+FN}$). To attenuate the influence of false negatives, we set β=0.5 to calculate F1_weighted_. The overall performance was evaluated using the area under the receiver operating characteristic curve (AUC) analysis. The statistical significance was set to *p* < 0.05.

## Results

### Dataset Characteristics

**Figure 3:**
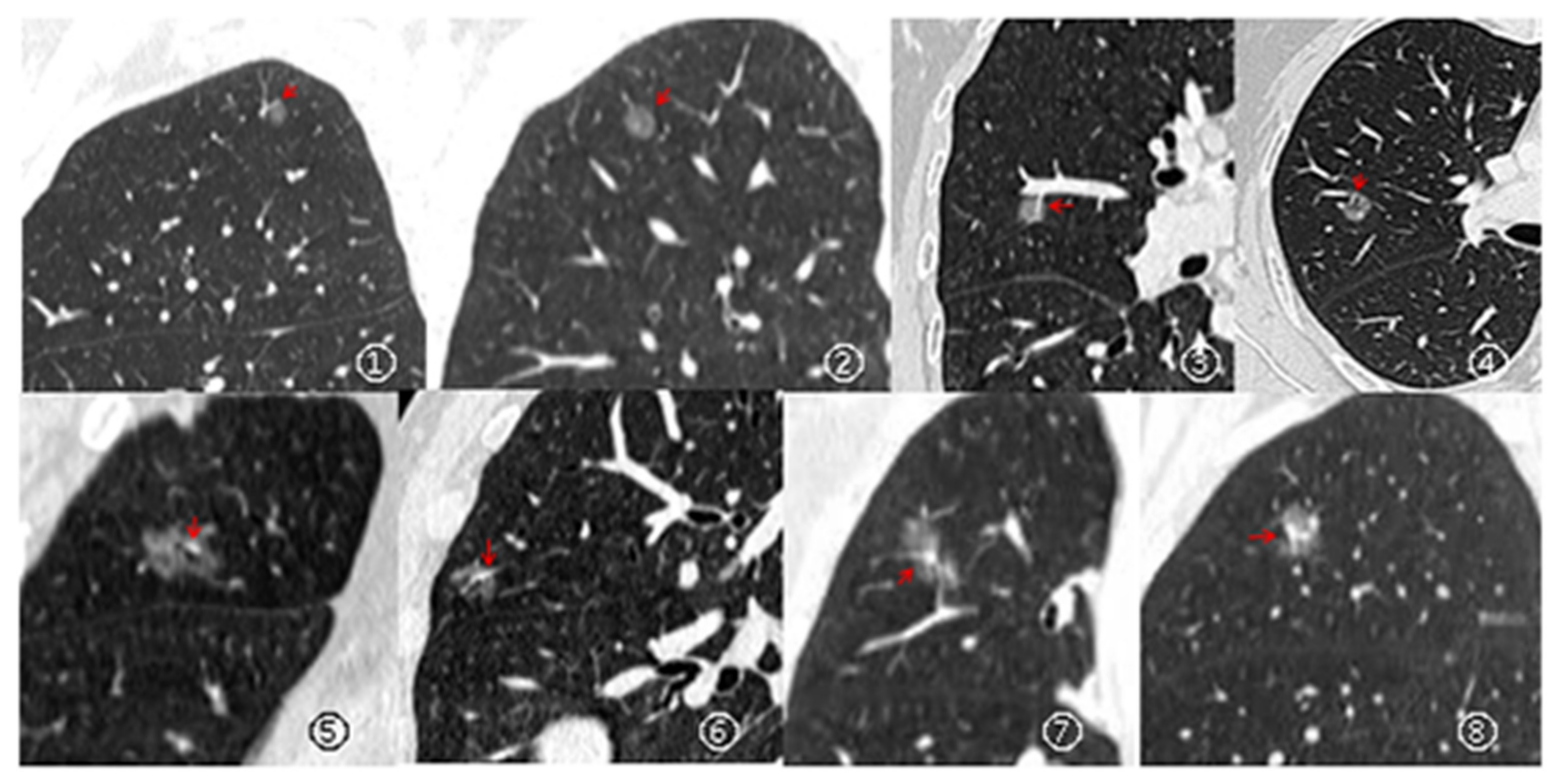
Types of relationships between GGNs and its vessels: Type I (pass-by, ①– ②), vessels passing by pGGNs without any detectable supply branches to the lesions; Type II (pass-through, ③– ④), vessels passing through the lesions without apparent morphological changes in traveling path or size; Type III (distorted/dilated, ⑤– ⑥), vessels within lesions that appear tortuous or rigid without an increase in amount; Type IV (complicated, ⑦– ⑧), more complicated vasculature than others described in the aforementioned types within pGGNs (e.g., coexistence of irregular vascular dilation and vascular convergence from multiple supplying vessels).

In this study, a dataset of 506 patients with 551 observations was established. Table 1 outlines the baseline patient data. There were six subjects with three nodules, 33 subjects with two nodules, and 467 subjects with only one nodule. Only 66 patients were presented with clinical symptoms. There were 24 (4.4%) non-neoplastic and 527 (95.6%) neoplastic (including atypical adenomatous hyperplasia, adenocarcinoma in situ, minimally invasive adenocarcinoma, and invasive adenocarcinoma) lesions. Twenty-four participants had a family history of carcinoma, and 30 participants had chronic obstructive pulmonary disease. There were 47 observations with G-V-R type I, 58 lesions with G-V-R type II, 64 with G-V-R type III, and 381 with G-V-R type IV. The CT images are shown in Figure 3. The age distribution of the patients was 56.5 ± 9.5 (mean ± SD) years and 56.3 ± 9.5 years for the training and validation set, respectively. A total of 327 (64.62%) women were included. The follow-up period between the training and validation sets was 43.6 ± 11.3 (mean ± SD) and 43.5 ± 12.1 months, respectively, and 229 patients maintained good compliance with medical advice.

**Table 1:** Clinical characteristics of patients between training set and validation set [means ± standard deviations; *n* (%)].

**Characteristics**	**Training set**	**Validation set**	***P***
**Gender**			
Male	75	104	0.668
Female	130	197	
Age (years)	56.5 ± 9.5	56.3 ± 9.5	0.844
**Family history of carcinoma**			
Yes	7	17	0.246
No	198	284	
**Clinical symptoms**			
Yes	27	39	0.772
No	178	262	
**Chronic obstructive pulmonary disease**			
Yes	10	20	0.293
No	195	281	
Period of follow-up (month)	43.6 ± 11.3	43.5 ± 12.1	0.990
**Compliance with medical orders**			
Yes	101	128	0.143
No	122	200	
**Distribution of nodules in patients**			
One	188	279	0.317
Two	16	17	
Three	1	5	
**G-V-R type**			
I	15	32	0.316
II	22	36	
III	23	41	
IV	163	218	
Size of pGGNs (mm)	13.96 ± 6.58	13.27 ± 5.82	0.195
**Lung adenocarcinoma spectrum**			
Non-neoplastic lesions	7	17	0.249
Neoplastic lesions	216	311	

*G-V-R, GGN-vessel relationship; pGGNs, pure ground-glass nodules; CT, computed tomography*.

**Table 2:** Summary of Lung-RADS version 1.1 of pGGN and its complementary Lung-RADS categories.

**Category**	**Lung-RADS 1.1**	**Complementary Lung-RADS 1.1**
		Stable or increased in size after two or more years follow-up
2	Size <30 mm	Type I of GVR and size <30 mm
3	Size ≥ 30 mm	Type I of GVR and size ≥ 30 mm; type II of GVR
4a		Any size with type III of GVR
4b		Any size with type IV of GVR
4x		Category 3 or 4 nodules with additional features or imaging findings that increases the suspicion of malignancy

*GVR, GGN-vessel relationship; Lung-RADS, lung imaging reporting and data system*.

**Table 3:** Summary of DL-based-cLung-RADS Version 1.1 used for the risk stratification management of pure ground-glass nodules.

**cLung-RADS**	**Risk scores**	**DL-based-cLung-RADS**
**1.1 category**	**of DL**	**1.1 category**
2	Low, middle, or high	2
3	Low	3
	Middle or high	4A
4A	Low	4A
	Middle or high	4B
4B	Low	4B
	Middle or high	4X
4X	Low, middle, or high	4X

*DL, deep learning; cLung-RADS, complementary lung imaging reporting and data system*.

### Performance of the CLung-RADS 1.1 and DL Models

Considering the training set, the DL model yielded a higher accuracy (91.03 vs. 84.30%), recall (99 vs. 97.9%), and F1_weighted_ (96.64 vs. 95.45%); nevertheless, it had a lower F1 score (89.40 vs. 91.31%) and MCC value (15.64 vs. 20.06%), compared to the cLung-RADS 1.1. The validation set was used to evaluate the performance of both cLung-RADS 1.1 and DL. The 90.55% validation accuracy value and 99% validation recall rate of the DL exceeded those of the cLung-RADS 1.1 (80.49%). Moreover, the DL model achieved a 94.99% F1 score, 95.27% F1_weighted_, and 95.45% precision rate, whereas cLung-RADS 1.1 achieved an 88.85% F1 score, a 93.54% F1_weighted_, and a 96.96% precision rate. However, the validation MCC value of cLung-RADS 1.1 was higher than that of the DL (19.43 vs. 2.73%). The AUC value of cLung-RADS 1.1 was higher than that of the DL in the training set (0.712 vs. 0.606, respectively) and validation set (0.676 vs. 0.561, respectively) set, as shown in Table 4.

**Table 4:** Comparison of diagnostic value for neoplastic lesions of lung nodule with cLung-RADS1.1, AI, and AI-based-cLung-RADS1.1.

	**Training set**			**Validation set**		
	**cLung-RADS1.1**	**DL**	**DL-based-cLung-RADS1.1**	**cLung-RADS1.1**	**DL**	**DL-based-cLung-RADS1.1**
TP	184	201	202	255	294	292
FP	3	5	3	8	14	8
FN	32	15	14	56	17	19
TN	4	2	4	9	3	9
Recall, %	97.9	99.0	98.1	96.6	99	97
Precision, %	98.4	97.57	98.54	96.96	95.45	97.33
MCC, %	20.06	15.64	32.43	19.43	2.73	37.15
F1 score (%)	91.31	89.40	95.96	88.85	94.99	95.58
F1_weighted_ (%)	95.45	96.64	97.49	93.54	95.27	96.62
Accuracy, %	84.30	91.03	92.38	80.49	90.55	91.77
AUC, (95% CI)	0.712 (0.489–0.934)	0.606 (0.366–0.845)	0.753 (0.526–0.980)	0.675 (0.529–0.820)	0.561 (0.409–0.712)	0.734 (0.585–0.884)

*FN, false negative; FP, false positive; TN, true negative; TP, true positive; MCC, Matthews correlation coefficient; DL, deep learning; cLung-RADS, complementary lung imaging reporting and data system; AUC, area under the curve; 95% CI, 95% confidence intervals*.

### DL-Based CLung-RADS 1.1 Model for Risk Stratification Management of Pulmonary GGNs

Considering the complementary performance in the risk management of GGNs, a novel DL-based cLung-RADS 1.1 model was developed to incorporate the additional information provided by the DL risk scores (Table 2). Regarding the training set, compared with both cLung-RADS 1.1 and DL, the DL-based cLung-RADS 1.1 model achieved the highest accuracy value (92.38%), F1 score (95.96%), F1_weighted_ (97.49%), and MCC value (32.43%). Considering the validation set, the DL-based cLung-RADS 1.1 model achieved excellent performance with a 97.33% precision, 91.77% accuracy value, 95.58% F1 score, and 37.15% MCC, although it had a lower recall rate than that of DL (93.89 vs. 94.53%). The DL-based cLung-RADS 1.1 model yielded the highest AUC value of 0.753 (0.526–0.980) and 0.734 (0.585–0.884) in the training and validation sets, respectively (Table 4).

## Discussion

In this study, we have developed a novel DL-based cLung-RADS 1.1 model to predict neoplastic lesions manifesting as GGNs on CT images. Our model was trained on and validated using data from patients with lung GGNs, and it demonstrated excellent performance in identifying both non-neoplastic and neoplastic GGNs, with a high degree of accuracy in both the training and validation sets. Our results indicate the potential value of using 3D-DCNNs for LC risk prediction and decision support for incidentally detected lung nodules. By ruling out CT scans with very high training and validation F1_weighted_ scores (99 and 96.62%, respectively), unnecessary workups, including imaging and invasive procedures, could be avoided in a significant number of patients.

Previous AI studies have focused on detecting and maximizing the proportion of correctly characterized cancers (i.e., high positive-predictive value or accuracy), and have shown promising results (14–18); however, the specificity of these tools is moderate. Wang et al. (18) used DL-based convolutional architecture for fast feature-embedding and ResNet-50 to detect GGNs, and reported an accuracy of 88% with an F-score of 0.891. In contrast, the framework of commercial DL in the present study was based on multi-stage 3D-DCNN algorithms, and FPRNet-101 was used for precise lung nodule classification, which achieved an excellent recall rate and F1 score, demonstrating superior performance over the algorithms used in Wang et al. 's study. Although the AUC value of DL in our study was lower than that of the previous AI scheme study (15) based on a DCNN (which used a residual learning architecture and batch-normalization technique). Our DL model exhibits consistently superior performance on a large-scale validation set with 328 GGNs in a clinical scenario.

Successful recognition of malignant GGNs can result in avoiding additional costs of multiple scans and can decrease patient anxiety. To improve the performance of DL in risk stratification of GGNs, the cLung-RADS 1.1 model, when used to manage national LC screening test data of China, yielded excellent performance in our previous study, achieving super sensitivity in predicting malignant nodules (13). Subsequently, the model has been used to complement the efficiency of DL in clinical scenarios. Therefore, a novel risk management model of GGNs using DL combined with cLung-RADS 1.1 was designed.This model achieved a superior precision rate, MCC, F1 score, and AUC value than those of the DL or cLung-RADS 1.1 models in our study, demonstrating the complementarity between DL and cLung-RADS 1.1. Furthermore, the persistently superior performance of the DL-based cLung-RADS 1.1 model was shown using a large sample size in our validation set.

Our study has several limitations. First, this was a single-center, retrospective study, and the percentage of neoplastic lesions was high (95.72%), which may have resulted in design and selection biases. Second, LDCT screening with the DL-based cLung-RADS 1.1 model does not avoid the risk of overdiagnosis or overtreatment because these pGGNs may be indolent and clinically insignificant. This can cause the rest of the patient's life to be subclinical. However, Henschke et al. (19) found that approximately 90% of diagnosed and untreated stage IA non-small cell LC (as small as 10 mm in diameter) had a malignant natural course and were fatal if not treated. A study by Caverly et al. (20) supports the importance of personalizing the harm/benefit assessment of LDCT LC screening for informing screening decisions, rather than providing uniform recommendations or withholding a recommendation for eligible patients. Therefore, further evaluation of the effectiveness of the DL-based cLung-RADS 1.1 model, with focus on pGGN cases, is recommended.

## Conclusion

Our results suggest that the proposed DL-based cLung-RADS 1.1 model is not only a better risk model for GGNs in identifying high-risk GGNs that require prompt intervention than cLung-RADS 1.1 or DL, respectively. It is also effective for the detection and diagnosis for LC screening in the some countries with large population sizes, which will reduce the frequency of CT scans to utilize the medical resources rationally or the patient's anxiety owing to long period follow-up. Therefore, it is beneficial to the public health services in China. However, the effectiveness of the model should be further verified in a multi-center study.

## Data Availability Statement

The raw data supporting the conclusions of this article will be made available by the authors, without undue reservation.

## Ethics Statement

The studies involving human participants were reviewed and approved by the Affiliated Tumor Hospital of the Zhengzhou University Medical Ethics Committee (Ethics Approval Number: 2021-KY-0022). Written informed consent for participation was not required for this study in accordance with the national legislation and the institutional requirements.

## Author Contributions

QM: had full access to all the data in the study, takes responsibility for the integrity of the data and the accuracy of the data analysis, and drafting of the manuscript. PG: statistical analysis. JD and JZ: administrative, technical, or material support. HG: supervision. All authors: concept, design, acquisition, analysis, or interpretation of data, and critical revision of the manuscript for important intellectual content. All authors contributed to the article and approved the submitted version.

## Funding

This study was received funding by the Science and Technology Project of Henan Province in China (No: 212102310744) and the Key project of Medical science and Technology of Henan Province in China (No: SBGJ20210257).

## Conflict of Interest

JD and JZ were employed by Yizhun Medical AI Co. Ltd. The remaining authors declare that the research was conducted in the absence of any commercial or financial relationships that could be construed as a potential conflict of interest.

## Publisher's Note

All claims expressed in this article are solely those of the authors and do not necessarily represent those of their affiliated organizations, or those of the publisher, the editors and the reviewers. Any product that may be evaluated in this article, or claim that may be made by its manufacturer, is not guaranteed or endorsed by the publisher.
